# Illuminating the Shadows: Innovation in Advanced Imaging Techniques for Myeloma Precursor Conditions

**DOI:** 10.3390/diagnostics15020215

**Published:** 2025-01-18

**Authors:** Kara I. Cicero, Rahul Banerjee, Mary Kwok, Danai Dima, Andrew J. Portuguese, Delphine Chen, Majid Chalian, Andrew J. Cowan

**Affiliations:** 1 Clinical Research Division, Fred Hutchinson Cancer Center, Seattle, WA 98109, USA; kcicero@fredhutch.org (K.I.C.); rahulban@uw.edu (R.B.); marykwok@fredhutch.org (M.K.); danaid@uw.edu (D.D.); aportugu@fredhutch.org (A.J.P.); 2 Division of Hematology and Oncology, School of Medicine, University of Washington, Seattle, WA 98115, USA; 3 Department of Radiology, University of Washington, Seattle, WA 98115, USA; dlchen7@uw.edu (D.C.); mchalian@uw.edu (M.C.)

**Keywords:** multiple myeloma, smoldering multiple myeloma, MGUS, imaging

## Abstract

Monoclonal gammopathy of undetermined significance (MGUS) and smoldering multiple myeloma (SMM), the asymptomatic precursors to multiple myeloma, affect up to 5% of the population over the age of 40. Bone involvement, a myeloma-defining event, represents a major source of morbidity for patients. Key goals for the management of myeloma precursor conditions include (1) identifying patients at the highest risk for progression to MM with bone involvement and (2) differentiating precursor states from active myeloma requiring treatment. Computed tomography (CT), magnetic resonance imaging (MRI), and positron emission tomography (PET)-CT with [^18^F]fluorodeoxyglucose (FDG) have improved sensitivity for the detection of myeloma bone disease compared to traditional skeletal surveys, and such advanced imaging also provides this field with better tools for detecting early signs of progression. Herein, we review the data supporting the use of advanced imaging for both diagnostics and prognostication in myeloma precursor conditions.

## 1. Introduction

Smoldering multiple myeloma (SMM) and monoclonal gammopathy of undetermined significance (MGUS) are asymptomatic precursor conditions that may progress to active plasma cell disorders defined by end-organ damage, most notably multiple myeloma (MM). Recent epidemiologic studies reveal that these precursor states are relatively common in the general population. A large, population-based study conducted in Iceland (iStopMM) found that 4.9% of the population over the age of 40 years had MGUS [1] and 0.53% had SMM [2].

The rate of progression from MGUS to malignancy is estimated to be 18% within 20 years and 28% within 30 years [3]. In contrast, SMM represents a more advanced precursor condition with a greater risk of progression to active MM: a 10% annual risk of progression to malignancy for the first 5 years, followed by a reduced risk of 2–3% per year thereafter [4].

Recent efforts have aimed to enhance risk stratification in MGUS and SMM. MGUS can be classified as low-, intermediate-, or high-risk based on the serum free light chain (sFLC) ratio, immunoglobulin isotype (IgG vs. other), and monoclonal protein quantification [5]. For SMM, the International Myeloma Working Group (IMWG) “20/2/20” model defines high-risk SMM by the presence of at least two of the following: bone marrow plasmacytosis ≥ 20%, a serum monoclonal protein ≥ 2 g per deciliter, and an sFLC involved–uninvolved ratio ≥ 20. The addition of high-risk cytogenetic abnormalities (i.e., t(4;14), t(14;16), gain(1q), and del(13q)) further refines risk within this model [6,7].

## 2. Role of Advanced Imaging in Myeloma Precursor Conditions

Advanced imaging plays a crucial role in the management of MGUS and SMM, as 40–50% of patients with myeloma precursor conditions ultimately progress to symptomatic myeloma without a notable increase in the monoclonal protein [8,9]. Therefore, advanced imaging is imperative to further characterize progression risk and early disease detection. Historically, plain radiographs of the axial and appendicular skeleton were utilized (the “skeletal survey” or “osseous survey”) to detect osteolytic bone disease. However, more advanced imaging modalities, such as whole-body low-dose computed tomography (WBLDCT), positron emission tomography with computed tomography (PET-CT) with [^18^F]fluorodeoxyglucose (FDG), and whole-body (wb) magnetic resonance imaging (MRI) with or without diffusion-weighted imaging (DWI), have since been shown to be superior to skeletal surveys [10,11]. In 2014, the IMWG even broadened the definition of active MM to include patients with more than one focal lesion on MRI even if no traditional “CRAB” criteria (hypercalcemia, renal disease, anemia, or lytic bone lesions) are present [12]. However, many uncertainties remain regarding best practice for these advanced imaging modalities. This review aims to provide guidance for the optimal utilization of WBLDCT, wbMRI, and PET-CT with respect to both the diagnosis and prognostication of myeloma precursor conditions.

## 3. Whole-Body Low-Dose Computed Tomography for Myeloma Precursor Conditions

Historically, screening for osteolytic lesions in myeloma precursor states relied on whole-body skeletal radiographs, which are easy to perform and widely accessible even in resource-limited healthcare systems. However, osseous surveys have significant limitations, most notably a lack of sensitivity, with lesions only visible after 30–50% of bone loss has occurred [11].

Compared to osseous surveys, WBLDCT demonstrates superior sensitivity for the detection of myeloma-related bone disease. In a retrospective analysis conducted by the IMWG, 146 patients with MM and 66 with SMM defined by osseous survey were evaluated; of those previously diagnosed with SMM, 22% were subsequently found to have osteolytic lesions on WBLDCT [13]. In another retrospective study, WBLDCT identified myeloma-related bone disease in 14 out of 40 patients (35%) who had been previously categorized as having a myeloma precursor condition by skeletal survey [14].

In a prospective study, investigators also demonstrated that serial monitoring with WBLDCT is effective at screening for early myeloma-related bone damage. Between July 2013 and March 2020, 100 patients with SMM underwent WBLDCT at the baseline, 1 year after diagnosis, and annually thereafter; among these patients, 10% were found to have disease progression based on WBLDCT [15].

Moreover, skeletal surveys are time-consuming, often taking 30 min to 1 h to complete, as multiple films of different bones must be taken individually. In contrast, WBLDCT typically takes only 5 to 15 min to perform and has largely replaced skeletal surveys as the minimum required imaging modality to rule out active MM, offering improved sensitivity and faster image acquisition [16].

### Summary—Whole-Body Low-Dose Computed Tomography for Myeloma Precursor Conditions

The IMWG has endorsed the use of WBLDCT as a screening tool for patients with high-risk MGUS and SMM [17], and the advantages of WBLDCT over conventional radiography are well-supported by the growing body of literature outlined above. However, there is a surprising lack of data on test performance metrics, such as sensitivity, specificity, and predictive values, for WBLDCT in those with MGUS. While the IMWG recommends WBLDCT screening for high-risk MGUS, this guidance appears to be based on expert consensus rather than empiric data. Nonetheless, WBLDCT offers superior sensitivity for detecting osteolytic lesions compared to conventional radiography and should therefore be considered standard practice for baseline imaging in high-risk MGUS. For SMM, however, both wbMRI and PET-CT have even greater sensitivity for detecting active disease, as discussed in the following sections.

## 4. Whole-Body Magnetic Resonance Imaging for Myeloma Precursor Conditions

Whole-body MRI (wbMRI) is a powerful tool for the early detection of myeloma bone disease. The presence of more than one focal lesion (FL) larger than 5 mm is considered a myeloma-defining event [12], as focal lesions represent aggregates of plasma cells which have the potential to develop into destructive lytic lesions [18]. Compared to CT, MRI excels at visualizing the bone marrow and soft tissues but is less effective at characterizing bone cortex [19].

Beyond its excellent sensitivity, MRI also boasts a high specificity, especially when accompanied by diffusion-weighted imaging (DWI). DWI can distinguish benign from malignant lesions, with malignant tumors often restricting diffusion more than benign tumors. Myeloma-related focal lesions alter the diffusion rate of water as measured by the apparent diffusion coefficient (ADC); lower ADC values (i.e., restricted diffusion) correlate with the degree of bone marrow infiltration [20].

### 4.1. Focal Lesions on Whole-Body Magnetic Resonance Imaging

In a study which evaluated 611 patients with MM, 52% of those with normal skeletal surveys had detectable FL on wbMRI [21]. Investigators then studied wbMRI in 149 individuals with SMM and found 42 patients (28%) to have FLs; the presence of more than one FL was associated with a median time to progression to symptomatic MM of 13 months [22]. Additionally, among 92 patients with SMM enrolled in the observational, prospective Southwestern Oncology Group (SWOG) S0120 trial, 7% had more than one FL on spine MRI, which was associated with an increased risk of progression according to univariate Cox proportional hazards (hazard ratio, HR, 4.17; *p* < 0.006) but was not an independent predictor of progression in a multivariate analysis adjusted for age, degree of bone marrow plasmacytosis, and monoclonal protein quantification [23]. Another analysis of 67 patients with SMM revealed that the presence of more than one FL on spine MRI was associated with an estimated time to progression of 15 months compared to 5 years for those without any FLs [24]. Accordingly, the IMWG updated the definition of active MM in 2014 to include the presence of more than one FL by wbMRI, for which treatment initiation is now recommended [12].

Data published since the revised definition of MM, however, highlight the need for more refined surveillance practices. In an analysis of 96 patients with SMM, investigators evaluated whether myeloma-related organ damage defined by the “CRAB” criteria (hypercalcemia, renal insufficiency, anemia, or lytic lesions) was reduced if treatment was started upon the updated “SLiM” criteria (bone marrow plasmacytosis over 60%, a free light chain ratio greater than 100, or MRI-defined FLs) [25]. After a median follow-up of 28 months, 22 patients progressed: 15 (68%) had end-organ damage, while the remaining 7 patients (32%) solely met the “SLiM” criteria. In nearly all patients, MRI FLs were detected alongside traditional osteolytic lesions at the time of progression; however not all patients had shown lesions in the wbMRI performed 6 months earlier. These findings highlight the significant unmet need to better identify and treat the two-thirds of patients who still present with myeloma-related organ damage despite regular MRI-based monitoring.

### 4.2. Focal Lesions: Volume, Kinetics, and Distribution

Tumor growth kinetics, as assessed by wbMRI in patients with SMM, were identified as a significant risk factor for progression to active MM in a retrospective analysis of 63 patients in Germany. Using the presence of more than one FL as an MM-defining event, the study reported a 2-year progression rate of 49.2%, a sensitivity of 48.3%, and a false-positive rate of 29.5%. Looking further, the authors discovered a correlation between the total tumor volume (TTV) and the time to progression. With a TTV threshold of 7220 cubic millimeters (mm^3^), they observed a 2-year progression rate of 80%, a sensitivity of 47%, and a false-positive rate of 8%.

The most effective prognostic indicator, however, was found to be the speed of tumor growth (SOG), calculated as the difference in TTVs divided by the time between consecutive wbMRI scans. In a study of 63 patients with SMM, SOG ≥ 114 mm^3^ per month exhibited a 2-year progression rate of 82.5%, a sensitivity of 63.2%, and a false-positive rate of 8.7% [26]. In another study, the analysis of consecutive wbMRI scans from 60 patients with more than one FL demonstrated that the growth rates of the largest FL and the fastest-growing FL emerged as strong predictors of progression, irrespective of the initial level of serum monoclonal protein [27].

It is important to note, however, that not all FLs will evolve into osteolytic lesions compatible with myeloma bone disease in the immediate future [25]. A recent analysis observed 29 participants with either MM or SMM who underwent concurrent MRI and CT scans and had at least one FL. The mean volume of FLs on MRI that correlated to osteolytic changes on CT was higher than for those without, but the difference was not statistically significant. Interestingly, FLs located in the axial skeleton were significantly more likely to have an osteolytic component than those in the extremities [28].

Thus, TTV and tumor growth kinetics on wbMRI appear to be far more powerful prognostic indicators than the revised IMWG criterion of more than one FL. Notably, however, this criterion was based upon studies that incorporated spine + pelvis imaging rather than whole-body imaging. Investigators subsequently analyzed wbMRIs in 147 patients with SMM to determine whether a broader anatomic coverage would alter the optimal number of FLs to define MM. “Whole-body” MRI protocols limited to the spine and the spine plus pelvis underestimated the presence of more than one FL, correctly identifying only 28% and 64% of cases, respectively. The 2-year risk of progression was 80% for patients with more than three FLs isolated to the spine and pelvis; however, the same risk of progression required more than four FLs identified on wbMRI [29]. Thus, the extent of anatomic coverage is crucial when assessing quantitative thresholds for FLs in relation to progression risk. Assuming universal access to wbMRI as part of MM screening, a higher cutoff of four FLs may more accurately predict progression to active MM.

### 4.3. Diffuse Infiltrative Pattern on Whole-Body Magnetic Resonance Imaging

A diffuse infiltration pattern on MRI has been investigated as a finding of interest in precursor conditions. This pattern is more frequently observed in SMM compared to MGUS, as MGUS more often demonstrates a minimal pattern of infiltration [30]. Some reports suggest that this diffuse infiltration pattern is linked with anemia, but studies have been inconclusive regarding its prognostic value for progression to MM [22,24,31,32].

In one study, 99 patients with plasma cell dyscrasias (20 with MGUS, 26 with SMM, and 53 with active MM) and 15 healthy controls were evaluated using wbMRI with DWI. Normal results were found in 28, moderate diffuse infiltration in 22, and severe diffuse infiltration in 49 participants. Although there was a statistically significant difference in infiltration patterns between the MGUS and MM groups, no significant difference was found between SMM and MM [33]. Additionally, the ADC, fat fraction, and T2* values were significantly higher in patients with MM compared to the MGUS and SMM groups and were associated with bone marrow plasmacytosis and hemoglobin levels across all patients. Another study found an inverse correlation between the diffuse infiltrative score (DIS) and the hemoglobin levels, but, when compared to other wbMRI parameters, such as the FL volume and growth dynamics, the severity of diffuse infiltration proved to be a less effective prognostic indicator for anemia development [27].

### 4.4. Summary—Whole-Body Magnetic Resonance Imaging for Myeloma Precursor Conditions

MRI has an established role in screening for myeloma bone disease in SMM, and the presence of FLs, even in the absence of osteolysis, is now considered a myeloma-defining event. However, even more important than the presence vs. absence of focal lesions appear to be the quantity of FLs, the kinetics of tumor growth, and the total tumor volume for predicting progression to active MM (Table 1).

## 5. [^18^F]Fluorodeoxyglucose (FDG) Positron Emission Tomography–Computer Tomography Imaging for Myeloma Precursor Conditions

Positron emission tomography–computer tomography with FDG (PET-CT) has a well-established role in the management of SMM, but there are limited data supporting the use of PET-CT as a screening tool for MGUS. While the 2003 IMWG criteria were vague about which imaging modality was preferred for diagnosing active MM, the 2014 IMWG criteria specified that the presence of one or more osteolytic lesions on PET-CT qualifies as a myeloma-defining event [12]. This specification was supported by a subsequent analysis of 188 patients with suspected SMM, 74 of whom had positive PET-CT and 25 of whom were followed longitudinally; those with underlying osteolytic lesions had a significantly higher rate of progression after 2 years compared to those without (87% vs. 61%) [34].

Historically, skeletal surveys were used in the evaluation of SMM. However, blinded radiology reviews of 79 patients referred for evaluation of SMM demonstrated that skeletal surveys were inferior to PET-CT for the assessment of myeloma bone disease, with a false-positive likelihood ratio of 31.3% and false-negative likelihood ratio of 85.7% [10]. Moreover, PET/CT may be helpful in identifying visceral extramedullary disease.

### 5.1. Patterns of Involvement on PET-CT: Diffuse vs. Focal, Without Osteolysis

Occasionally, abnormal FDG uptake on the PET component is detected in patients with SMM, without corresponding osteolysis on the CT component. The optimal management of these patients is unclear, as FDG uptake alone is not considered an MM-defining event under the IMWG diagnostic criteria [12]. In a large prospective analysis of 120 patients with SMM from several European centers, 16% of patients were found to have focal FDG uptake on PET without osteolysis on CT, reflecting a 3-fold risk of progression to active MM (HR 3.00, 95% CI 1.58–5.69); the median time to progression for patients with focal uptake was 1.1 years compared to 4.5 years for those without focal uptake [37].

The degree of hypermetabolism on PET-CT is less understood, but attempts have been made to correlate certain uptake patterns with plasma cell involvement of the bone marrow. For example, an analysis of the standardized uptake value (SUV) of the L4 vertebral body in 65 patients with SMM did not correlate with bone marrow plasma cell concentration as determined by core biopsy or flow cytometry [38]. In contrast, the intensity of bone involvement (IBI) score is a quantitative image analysis of generalized bone marrow uptake on PET-CT and has been found to correlate with hemoglobin values in patients with SMM; thus, the IBI is thought to be an objective, non-invasive measure of bone marrow plasmacytosis [39]. However, this type of analysis is not widely available, limiting its clinical utility.

### 5.2. The Role of Texture Analysis in FDG PET-CT Imaging of Smoldering Multiple Myeloma

With advancements in image processing, the texture analysis of PET-CT can now be used to evaluate the heterogeneity of intra-tumoral uptake in various malignancies [35]. A deeper understanding of FDG uptake heterogeneity within tumors could provide valuable insights into the underlying disease biology. Applied to patient-level imaging, this detailed analysis could offer a more nuanced understanding of a patient’s disease characteristics beyond what is captured in standard imaging reports [35]. Texture features are generated using mathematical algorithms applied to extracted imaging data and can be categorized into first-order (i.e., intensity, shape, or volume) and higher-order textural features (i.e., advanced quantitative metrics which assess spatial distribution and relationships of intensities within an image).

In one of the few studies examining higher-order texture analysis (TA) in patients with SMM, researchers analyzed 45 patients with SMM who underwent PET-CT. Shape, second-order, and higher features in standard regions placed in the thoracic and lumbar spine, iliac crests, and femoral diaphyses were significantly associated with time to progression to symptomatic MM in this cohort. The strongest associations with time to progression were found with higher-order TA features from the iliac crest and femoral diaphysis. In contrast, texture parameters derived from the spine showed no significant association with progression [40].

### 5.3. Summary—PET-CT for Myeloma Precursor Conditions

PET-CT plays an important role in screening for myeloma bone disease in patients with myeloma precursor conditions. Beyond detecting osteolytic lesions, PET-CT provides valuable insights into the risk of progression to MM, such as focal uptake without osteolysis or diffuse bone uptake (Table 1). Emerging radiomics techniques hold promise to further enhance the utility of PET-CT in assessing precursor conditions.

## 6. Discussion

**Figure 1:**
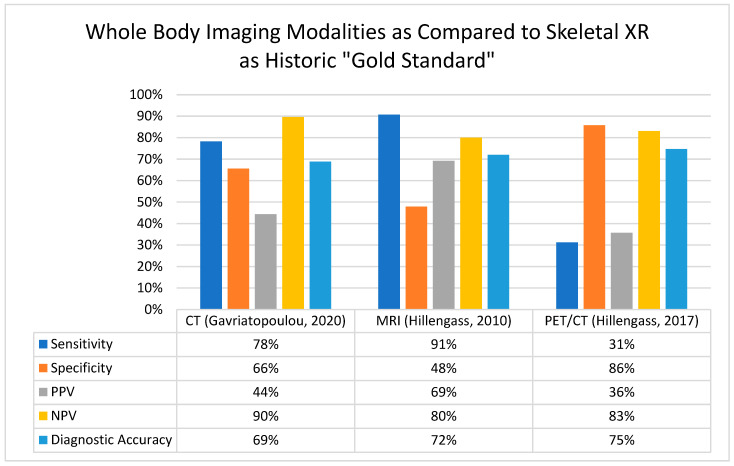
Comparison of whole-body imaging modalities for the detection of myeloma bone disease [11,13,20]. Abbreviations: CT, computed tomography; MRI, magnetic resonance imaging; NPV, negative predictive value; PET-CT, positron emission tomography/computed tomography; PPV, positive predictive value; and XR, X-ray.

Over the past 20 years, the field of myeloma has witnessed significant improvements not only in survival rates due to more effective therapies, but also in the development of advanced diagnostic tools. Figure 1 summarizes the comparisons of sensitivity, specific, positive predictive value, negative predictive value, and overall diagnostic accuracy between whole-body CT, MRI, and PET/CT, compared to the historic standard of care, the X-ray. These advancements have facilitated the earlier identification of patients at high risk of disease progression and improved prognostication, allowing for more adequate monitoring for those in need, as suggested by Table 2.

In recent years, wbMRI with DWI has become a valuable technique for monitoring for myeloma bone disease, particularly in patients with SMM. While the 2014 IMWG criteria included the presence of more than one FL as a diagnostic criterion for MM, it is now evident that other imaging features also hold considerable importance. These include total lesion volume [27,28], growth kinetics [26,27,30], geographic distribution of lesions [29], and pattern of involvement (diffuse vs. focal) [30] (Table 1).

Notably, there remains a lack of research focused on advanced imaging for screening patients with MGUS. Although many of the studies reviewed include subsets of patients with MGUS, there are surprisingly few data to directly support the routine use of advanced imaging in this population. The current IMWG guidelines recommend the use of WBLDCT only for high-risk MGUS (REF), a recommendation based largely on expert opinion rather than robust data. Given the prevalence of MGUS in individuals over the age of 50, further research is needed to better assess the utility of bone imaging in this group, with a particular focus on its positive predictive value. In practice, the distinction between MGUS and SMM is often unclear at the time of initial diagnosis, leading physicians to order both a bone marrow biopsy and WBLDCT for convenience. However, this raises concerns about incidental findings and radiation exposure, which should be considered.

Looking ahead, research in advanced imaging for myeloma precursor conditions could benefit from insights gained from targeted imaging approaches in other malignancies. Recently, there has been progress in developing BCMA-targeted PET imaging, with previous efforts exploring CD38-targeted PET as well [36,41,42]. These modalities hold promise for better identifying patients at the highest risk of progression and pinpointing lesions more likely to develop into osteolytic bone disease. Ultimately, more translational research is needed, integrating biopsies or genomic/TME analyses, to enhance our understanding of the biology and progression risk in patients with FLs identified through imaging.

## Figures and Tables

**Table 1:** Prognostic indicators on whole-body imaging for progression to symptomatic myeloma.

	Prognostic Indicator	Median TTP	2-Year Progression Risk	Univariate HR (*p*-Value or 95%CI)
***MRI***	Quantity of FL			
	>1 on whole body [21]	1.1 years	49.2%	4.05 (<0.001)
	>1 on axial [23]	1.3 years		
	>4 on whole body [28]		80%	
	>3 on axial [28]		80%	
	Total tumor volume of FL [25]			1.48 (0.001)
	TTV ≥ 7220 mm^3^ [24]		80%	
	Speed of growth [25]			1.99 (0.003)
	Increase ≥ 114 mm^3^/mo [24]		85.2%	
	Diffuse infiltration [21,25,34]			1.77–3.14 (<0.001; 1.2–6.5)
***PET/CT***	Focal FDG uptake without osteolysis [34]	1.1 years		3.0 (1.6–5.7)
	SUV of L4 [35]			1.698 (0.955–3.021)
	Texture [36]			
	Second order Femoral diaphysis	1.1 years		
	Higher features L2–4	1.1 years		

**Table 2:** Suggested whole-body imaging for myeloma precursor states.

Low-risk MGUS	Imaging only as clinically indicated
High-risk MGUS	Low-dose whole-body CT for the baseline; thereafter, as clinically indicated or upon progression of myeloma labs
Low-risk SMM	Alternating DW MRI and PET/CT annually ×5 years from diagnosis or as clinically indicated; thereafter, as clinically indicated or upon progression of myeloma labs
High-risk SMM	Alternating DW MRI and PET/CT q6 mo ×5 years; thereafter, as clinically indicated or upon progression of myeloma labs or concern for evolving disease (e.g., declining hemoglobin, increasing M spike kinetics) [8,9]

## Data Availability

Not applicable.
